# Postoperative complications and nutritional status between uncut Roux-en-Y anastomosis and Billroth II anastomosis after D2 distal gastrectomy: a study protocol for a multicenter randomized controlled trial

**DOI:** 10.1186/s13063-019-3531-0

**Published:** 2019-07-12

**Authors:** Shi Chen, Dong-Wen Chen, Xi-Jie Chen, Yi-Jia Lin, Jun Xiang, Jun Sheng Peng

**Affiliations:** 1 The Sixth Affiliated Hospital, Sun Yat-sen University, No 26, YuanCun ErHeng Road, TianHe District, Guangzhou, 510655 China; 2 Guangdong Provincial Key Laboratory of Colorectal and Pelvic Floor Diseases, The Sixth Affiliated Hospital, Sun Yat-sen University, Guangzhou, China

**Keywords:** Uncut Roux-en-Y anastomosis, Billroth II anastomosis, D2 distal gastrectomy, Nutritional status

## Abstract

**Background:**

Gastric cancer is the fourth most common malignant disease worldwide, with lower one-third gastric cancer the most common type. Distal gastrectomy with D2 lymph node dissection was recommended as a standard surgery for distal gastric cancer patients. However, some controversy remains about the anastomosis of the residual stomach and the intestine. The objectives of this trial are to test the hypothesis that uncut Roux-en-Y anastomosis can reduce postoperative complications and improve nutritional status more effectively than Billroth II anastomosis in gastric cancer patients after D2 gastrectomy.

**Methods/design:**

This multi-center, prospective, phase III, randomized controlled trial will compare the efficacy of uncut Roux-en-Y anastomosis versus Billroth II anastomosis in phase I–III patients with initial treatment of radical distal gastrectomy. Patients will be randomized to undergo either the intervention (uncut Roux-en-Y anastomosis) or the control (Billroth II anastomosis). We will recruit 832 patients who meet the trial eligibility criteria and will follow the patients after surgery to observe postoperative complications and nutrition status for 5 years. The primary assessment indices of the study are reflux gastritis, esophagitis, bile regurgitation, and anastomotic ulcer. The secondary assessment indices are nutritional status, quality of life, perioperative complications, overall survival rate, and others. When the number of cases reaches 400, an interim analysis will be performed to identify any evidence of definite superiority of the experimental intervention.

**Discussion:**

We aim to test the hypothesis that uncut Roux-en-Y anastomosis can reduce postoperative complications and improve nutritional status more than Billroth II anastomosis in gastric cancer patients after D2 gastrectomy. The results of the trial will contribute to the best evidence on which to base the reconstruction of distal gastrectomy.

**Trial registration:**

Chinese Southern Gastric Cancer Conference CSGC002 Trial. ClinicalTrials.gov, NCT02763878. Registered on 5 May 2016.

**Electronic supplementary material:**

The online version of this article (10.1186/s13063-019-3531-0) contains supplementary material, which is available to authorized users.

## Background

Gastric cancer remains the fourth most common malignant disease and is the second leading cause of cancer-related deaths worldwide, although the mortality is decreasing in many countries, such as in North America and Eastern Europe [1, 2]. Lower one-third gastric cancer is the most common type of gastric cancer, accounting for more than 50% of cases [3]. For these patients, distal gastrectomy with D2 lymph node dissection was recommended as the standard surgery [4].

To date, we have three main techniques for anastomosis between the residual stomach and the intestine—Billroth I anastomosis, Billroth II anastomosis, and Roux-en-Y anastomosis—among which Billroth II and Roux-en-Y anastomosis have been the most used [5–7]. However, some controversy remains regarding anastomosis of the residual stomach and the intestine, as each anastomosis has its own advantages and disadvantages [8–10]. Billroth I anastomosis is simple for surgeons to perform, but the procedure can only be performed on specific patients as it is likely to cause high tension for the anastomosis and reflux diseases, including reflux gastritis and esophagitis [11, 12]. Billroth II anastomosis is also simple to perform and is associated with lower anastomotic tension, but it is possible to cause bile reflux gastritis, esophagitis, or dumping syndrome [13]. Roux-en-Y anastomosis has been explored for use in distal gastrectomy for the past 20 years. It can partly resolve the reflux diseases that always occur after Billroth I and Billroth II anastomosis [14, 15]. However, Roux-en-Y anastomosis is more complex than the other two kinds of anastomosis; in this procedure, the small intestine is cut off to connect the residual stomach with the proximal jejunum, interrupting the continuity of the small intestine and influencing nerve impulse transmission [16, 17]. In theory, an ideal gastrointestinal reconstruction after distal gastrectomy would have the following characteristics: continuity of the digestive tract, less occurrence of postoperative syndromes, and easy acceptance by surgeons.

**Fig. 1:**
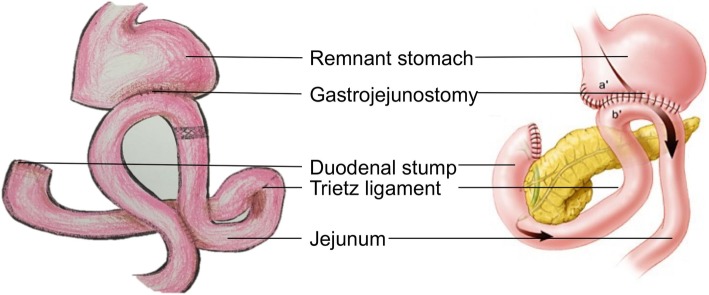
The general view of surgical procedures. The left one is Uncut Roux-en-Y anastomosis, while the right one is Billroth II annastomosis

Uncut Roux-en-Y anastomosis was first reported by Van Stiegmann and Goff in 1988 [18]. In 2005, Uyama et al. [19] first reported laparoscopy-assisted uncut Roux-en-Y reconstruction after distal gastrectomy. The anastomosis method of the uncut Roux-en-Y is shown in Fig. 1. In theory, this anastomosis has an anti-reflux function and can eliminate the occurrence of Roux stasis syndrome [20, 21]. Moreover, this anastomosis preserves the continuity of the gastrointestinal tract, maintains nerve impulses generated from the normal pacemaker of duodenum to distal jejunum, and prevents ectopic pacemakers [22, 23]. Kim et al. first reported that uncut Roux-en-Y anastomosis could help to reduce the occurrence of reflux gastritis and bile reflux [24]. In the retrospective study of Liang and colleagues [25], comparing the procedure with Billroth II anastomosis, the occurrence of anastomotic ulcer and reflux gastritis after gastrectomy was lower with uncut Roux-en-Y anastomosis. Thus, the uncut Roux-en-Y is controversial but promising. However, there have been few reports on this anastomosis, most of which were either retrospective studies or had small sample sizes. No studies of postoperative nutritional status have been reported for gastric cancer patients after D2 gastrectomy with uncut Roux-en-Y anastomosis. We propose to develop this multi-site, prospective clinical trial to investigate the advantages of uncut Roux-en-Y anastomosis compared with Billroth II anastomosis.

### Trial objectives

The objectives of this trial are as follows:

1. To test the hypothesis that uncut Roux-en-Y anastomosis can reduce postoperative complications and improve nutritional status for gastric cancer patients after D2 gastrectomy compared to Billroth II anastomosis
2. To provide class-one evidence for the reconstruction of distal gastrectomy

## Methods/design

### Trial design

**Fig. 2:**
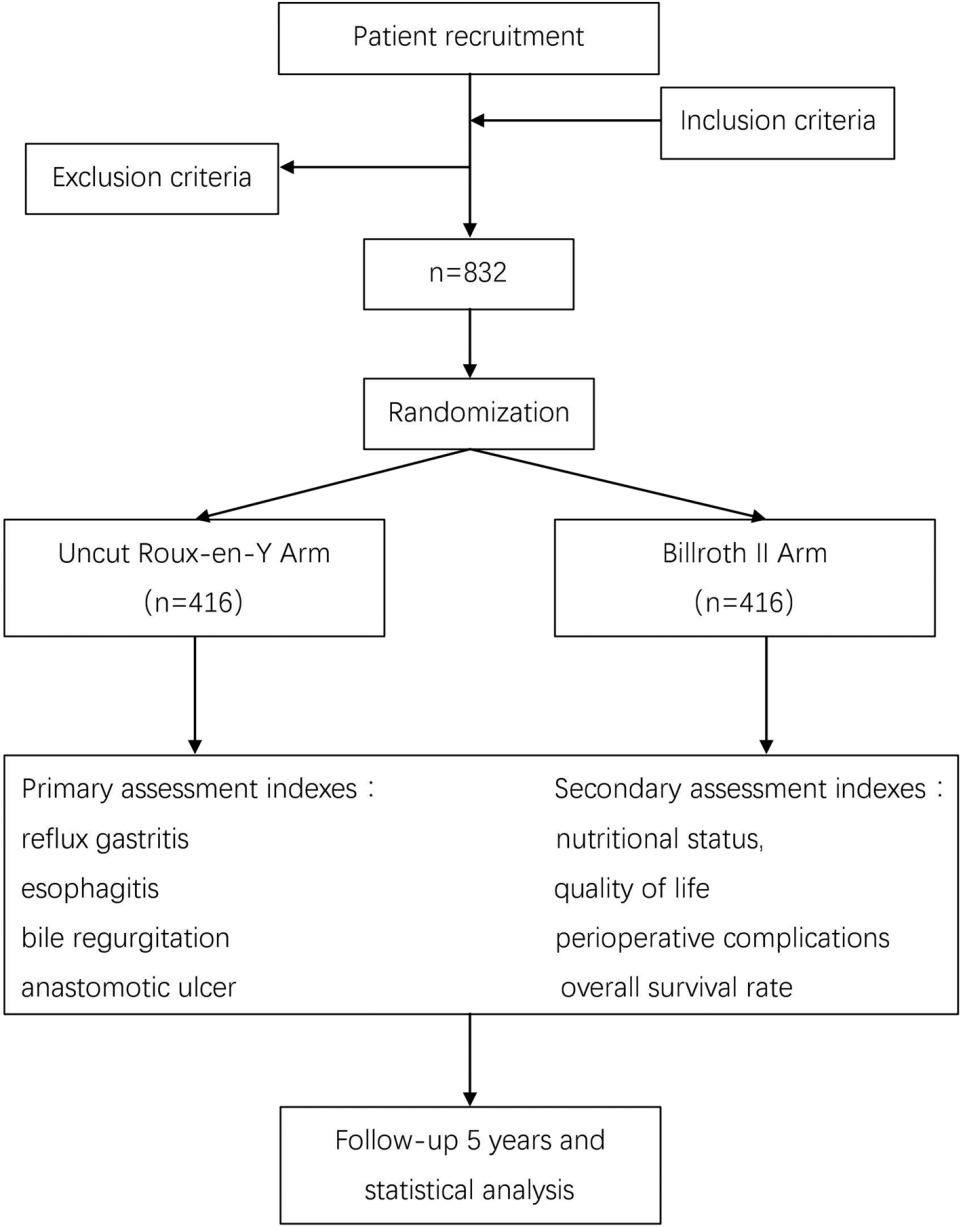
Flow chart of the study

**Fig. 3:**
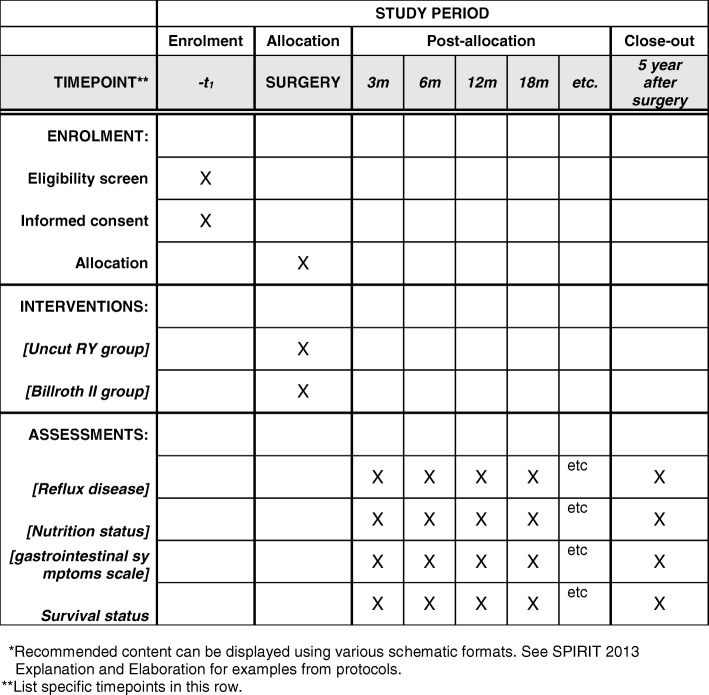
Example template of recommended content for the schedule of enrolment, interventions, and assessments

This is a multi-center, prospective, phase III, randomized controlled trial, supported by the China Southern Gastric Cancer Cooperation (CSGC). The Sixth Affiliated Hospital of Sun Yat-sen University associated with the other eighteen hospitals in Southern China is responsible for this trial. Patients from the coordinating centers who satisfy the pre-specified criteria will be included and randomly divided into two groups: an experimental group undergoing uncut Roux-en-Y anastomosis and a control group undergoing Billroth II anastomosis. We will follow the patients after surgery to observe postoperative complications and nutrition status for 5 years. The study flow chart is shown in Fig. 2. An interim analysis will be undertaken when 400 eligible cases are enrolled, and we will subsequently evaluate whether the trial should be continued or terminated depending upon the results of the interim analysis. The example template of recommended content for the schedule of enrolment, interventions and assessments is shown in Fig. 3.

### Ethics

Ethics approval for this study was granted on 18 April 2016 by the Ethics Committee of The Sixth Affiliated Hospital of Sun Yat-sen University (reference number 2016ZSLYEC-009). The study protocol, patient information sheets, and informed consent forms were approved by the ethics committee. Informed consent will be obtained from all participants. Participants’ real names will not appear on the relevant reports of this trial in order to protect their privacy.

### Study setting

Participants are to be recruited from The Sixth Affiliated Hospital of Sun Yat-sen University, Sun Yat-sen University Cancer Center, The First Affiliated Hospital of Sun Yat-sen University, The Third Affiliated Hospital of Sun Yat-sen University, Guangdong General Hospital, Guangdong Chinese Traditional Medicine Hospital, NanFang Hospital of Southern Medical University, ZhuJiang Hospital of Southern Medical University, Cancer Center of Guangxi Medical University, General Hospital of Guangzhou Military Command of PLA, Hainan General Hospital, YueBei People’s Hospital, Shenzhen People’s Hospital, Meizhou People’s Hospital, Affiliated Hospital of Guangdong Medical University, Cancer Hospital of Shantou University Medical College, Shantou Central Hospital, Cancer Center of Guangzhou Medical University, and The First Affiliated Hospital of Guangzhou Medical University. All patient visits will be performed in the hospital where the participants are recruited. Data management and trial coordination will be performed at The Sixth Affiliated Hospital of Sun Yat-sen University. This study was adopted into the Chinese Southern Gastric Cancer Conference as trial CSGC002.

### Sample size

Our study is designed as a superiority trial whose primary outcome is postoperative reflux gastritis or not. Current experience suggests the predicted rate of postoperative reflux gastritis will be 8% for the uncut Roux-en-Y anastomosis group and 12% for the control group of Billroth II anastomosis. The difference test was used to estimate the sample size with 80% test power and a 5% significance level. Accounting for a 15% drop-out rate and the 1:1 allocation ratio, the total number of patients required per group is 416 (832 in total), which was calculated using the software PASS version 11.0. Considering a completion rate of 75% for each coordinating center, 1040 envelopes were designed for random allocation. The number of cases will be increased if necessary according to the results of the interim analysis.

### Inclusion criteria

To be enrolled, participants must be phase I–III patients with initial treatment of radical distal gastrectomy and satisfying the following inclusion criteria:

1. Histologically proven stage I–III gastric cancer, evaluated as radically resectable
2. No synchronous or metachronous cancers
3. Patients have signed informed consent forms
4. Age 18–75 years old
5. No malfunction of cardio-pulmonary, liver, and kidney, ECOG score 0–1
6. No emergency surgery needed

### Exclusion criteria

Patients will be excluded according to the following criteria:

1. Pregnant or lactating women
2. Distant metastasis to the liver, lung, bone, supraclavicular lymph nodes, pelvic, or ovarian species and peritoneal dissemination
3. Ascites or cachexia
4. Suffering from other serious diseases, including cardiovascular, respiratory, kidney, or liver disease, poorly controlled hypertension or diabetes
5. Participation in other clinical trials 4 weeks before the enrollment of this trial or still participating in other trials
6. Mental illness
7. Surgical history whose influence has not been eliminated
8. History of another gastric or esophageal malignancy, including stromal tumor, sarcoma, lymphoma, and carcinoid
9. Active infection with a fever of over 38°C
10. Poor compliance
11. Not suitable for this trial because of other clinical or laboratory conditions determined by the researchers

### Elimination criteria

1. Efficacy evaluation of primary outcome: if data for more than three of a total of six time-points (6 months, 1 year, 2 year, 3 year, 4 year, and 5 year) missed for the patient.
2. Did not evaluate the efficacy according to the predetermined plan.

### Recruitment

Patient recruitment from the centers began on 1 January 2016 and will be finished in December 2018 or when the required number of participants has been obtained, whichever is sooner. Eligible patients will be informed about the trial, allowed to ask questions about the study as well as treatments, and invited to participate in the trial.

### Randomization

After completing an initial assessment and signing an informed consent form, participants will be randomized into two groups: the experimental group undergoing uncut Roux-en-Y anastomosis and the control group undergoing Billroth II anastomosis.

The randomization is undertaken only by the qualified researchers. A secure randomization routine will give each patient an identification number. This number must be recorded in the randomized list together with the randomized data. The completed list should be signed by the investigators and submitted to the data center along with the patients’ initial information. Patients’ identification numbers should be marked in all the relevant reports.

During the randomization, allocation concealment should be maintained. All the patients will be randomized into groups in a 1:1 ratio, and neither surgeons nor patients will be informed as to the randomization arm before surgery. Once D2 gastrectomy has been performed, the randomization arm of the patient will be revealed and the corresponding anastomosis will be performed.

### Intervention

The surgical procedures of the uncut Roux-en-Y arm and Billroth II arm are as follows.

#### Uncut Roux-en-Y arm

After distal gastrectomy, duodenal stump closure, side-to-side anastomosis is performed on the remnant stomach and jejunum, 25 cm from the ligament of Treitz. Then, side-to-side anastomosis between the jejunum approximately 35 cm from gastrojejunostomy and the jejunum approximately 5 cm from the ligament of Treitz is performed. The intestinal cavity on the input less than 5 cm from the loop gastrojejunostomy anastomosis is closed using uncut closure devices.

#### Billroth II arm

After distal gastrectomy and duodenal stump closure, the investigators first perform remnant stomach and upper jejunum side anastomosis. Then, they choose the jejunum approximately 25 cm from the ligament of Treitz, to the premenstrual colon using a disposable cutting closure (or tubular stapling) in the rear wall of the stomach and jejunum anastomosis; common opening is closed with (barbed wire) hand-stitching. Subsequently, the steps are the same as group A (uncut Roux-en-Y arm).

### Uniformity in surgical performance

Assuring uniformity in surgical performance is the key point for our multi-center clinical trial. Therefore, the surgeons definitely should be trained and the operative procedures monitored. It is worth mentioning that the surgeons should at least have clinical experience in conducting radical gastrectomy for more than 3 years or more than 100 cases.

Before the trial, the leading center will appoint qualified surgeons to train the other surgeons in the coordinating centers. It is relatively easy for them to master the new technique for the reason that the uncut Roux-en-Y anastomosis is based on Billroth II anastomosis with the addition of the side-to-side anastomosis between the jejunum approximately 35 cm from gastrojejunostomy and the jejunum approximately 5 cm from the ligament of Treitz and closure of the intestinal cavity on the input less than 5 cm from the loop gastrojejunostomy anastomosis using uncut closure devices.

In particular, it is required to take pictures of the surgery field including the lymph node dissection area and the anastomosis field in order to make sure that the performance is qualified. These pictures will be uploaded to the database and checked by the data managers. If the operation does not achieve the standard of either D2 gastrectomy or corresponding anastomosis, the case will be rejected.

### Outcome measures

All patients enrolled in this trial will be followed up regularly at stated intervals after the surgery for an average of 5 years. During the follow-up visits, patients will undergo endoscopy, laboratory tests, and radiography if necessary as described in Table 1.

**Table 1:** Summary of the follow-up visit schedule and assessed parameters at each time point

	Preoperative	3 M	6 M	9 M	12 M	18 M	24 M	36 M	48 M	60 M
Endoscopy	*	**	*		*		*	*	*	*
Laboratory	*	*	*		*		*	*	*	*
CT scan	*	*	*		*		*	*	*	*
Upper digestive tract radiography	**	*	*		*		*	*	*	*

*Evaluation at the designated time point, **evaluation if necessary

The primary assessment indices of the study are reflux gastritis, esophagitis, bile regurgitation, and anastomotic ulcer. Endoscopy will be used to help with standardized assessment of these indices during the follow-up visits. Full details for the Los Angeles classification of the severity of reflux esophagitis are shown in Table 2.

**Table 2:** Los Angeles classification of the severity of reflux esophagitis

Grade	Manifestation
A	One (or more) mucosal break(s) no longer than 5 mm that does not extend between the tops of two mucosal folds
B	One (or more) mucosal break(s) more than 5 mm long that does not extend between the tops of two mucosal folds
C	One (or more) mucosal break(s) that is continuous between the tops of two or more mucosal folds but which involve(s) less than 75% of the circumference
D	One (or more) mucosal break(s) that involve(s) at least 75% of the esophageal circumference

The secondary assessment indices are nutritional status, quality of life, perioperative complications, overall survival rate, and others. Nutritional status includes body mass index (BMI), hemoglobin, albumin, total protein, pre-albumin, transferring, and other indices such as appetite and Onodera’s prognosis nutritional index (PNI). Quality of life will be assessed by the gastrointestinal symptom rating scale (GSRS) and Visick score (shown in Additional file 1: Tables S1 and S2). Perioperative complications occurring within 30 days after the surgery include anastomotic fistula and anastomotic stenosis. Overall survival rates for 1, 3, and 5 years after the surgery as well as recurrence and metastasis rates will be calculated. Other indices include surgery time, intraoperative blood loss, length of hospital stay, and hospital cost.

All the results of the outcome measures will be recorded at the data center. Only follow-up data of patients without recurrence after 1 year of surgery will be analyzed at the end of the study.

### Data management

#### Data collection

All the information should be honestly and precisely recorded in a timely manner in the case report form (CRF) or CRF software. Researchers are required to record the patients’ information in the CRF according to the program of the trial. The research center will appoint supervisors to examine the completeness and accuracy of the CRF and to guide the researchers in making changes or additions if necessary. The CRF is submitted to data management by the region supervisor, with one copy remaining in the research center and the other one as a working attachment for the supervisor. Reliable data managers for medicine will input the data from the CRF to the database. The CRF should be filled in by a specific person assigned by the coordinating center and will not make sense until it is signed by the chief in the corresponding center. The center in charge will regularly check and collect the CRF in the coordinating centers. The database generated by software will be transmitted over the network to the center in charge.

At the end of the trial, each coordinating center should submit a summary of their own according to clinical summary standards. The leading center will arrange all the forms and make conclusions. An overall summary according to clinical summary standards is required.

#### Case report form

In the trial, observation or examination items need to be recorded in the CRF. The contents of the CRF should be identical to the original materials. In the CRF, the results calculated from the original materials should be able to be back-dated.

The CRF must be filled in with the following criteria satisfied:

1. Use black pen or black ball-point pen to fill in data.
2. Data from patients who have signed informed consent and met inclusion criteria, but who are subsequently judged to fail to meet inclusion criteria after treatment or even without drug treatment, should be recorded in the CRF.
3. Draw on the original record with horizontal lines when making corrections (correction fluid is not allowed). Make sure the original record is identifiable and then sign and date the correction.
4. For items not yet examined, “ND” should be marked in the CRF.

#### Database management and quality control

Measures are taken to achieve quality control. Data items of the CRF will be input into the database. Text items can only be checked manually at the first time they are input. Subsequently, data managers will systematically check the information in the database. The database will be locked when completed without errors. Any changes in the database can only be achieved with the joint written consent from clinical research leaders, statisticians, and data managers.

### Data analysis

#### Interim analysis

When the number of cases reaches 400, an interim analysis will be performed to identify any evidence of definite superiority of the experimental intervention. The interim report includes the following content:

1. The number of the enrolled patients and the planned time for completion
2. The number of cases with compliance and non-compliance
3. Assessment of quality of the materials according to the submission time, completeness, and accuracy of the materials
4. Preliminary analysis of treatment efficacy, including nutritional status, quality of life, and rate of complications
5. Incidence and classification of adverse reactions

The central center will regularly report to all the coordinating centers according to the above information. If necessary, this study will be adjusted or terminated depending upon the results of the interim analysis.

#### Statistical analysis

Statistical analysis planning was formulated by health statisticians as well as key researchers. We will use SPSS or SAS to analyze the data. For survival analyses, Kaplan-Meier curves and log rank tests will be used. The Cox proportional hazards model will be used for prognosis analysis. Categorical variables will be analyzed by Pearson’s χ^2^ test or Fisher’s exact test, and continuous variables will be evaluated by Student’s *t*-test or an appropriate non-parametric method as required. The level of statistical significance will be set at 5%.

#### Subgroup analysis

Subgroup analysis will be as follows: 1) preoperative NRS2002 scoring (< 3 group and ≥ 3 group); and 2) pathological TNM staging (stage I, stage II, and stage III) to precisely analyze the advantage of uncut Roux-en-Y anastomosis.

## Discussion

In recent years, gastric cancer has remained the fourth most common malignant disease worldwide, and lower one-third gastric cancer is the most common type [1–3]. Distal gastrectomy with D2 lymph node dissection has been thought to be the standard surgery for these patients [4]. However, reconstruction of distal gastrectomy remains controversial; Billroth II anastomosis has gained widespread popularity for its simplicity and lower risk of anastomotic tension. In addition, Billroth II anastomosis may cause bile reflux gastritis, esophagitis, or dumping syndrome [13]. Therefore, the preferred way to perform reconstruction after distal gastrectomy is still debated. Uncut Roux-en-Y anastomosis is another method of reconstruction with an anti-reflux function as well as the ability to eliminate the occurrence of Roux stasis [20, 21]. However, it requires further investigation to have an impact in clinical practice.

Many investigators have evaluated the anastomosis after distal gastrectomy by randomized controlled trials [19, 26–29]. However, most of these studies were conducted with a small population at a single center. Moreover, there are no prospective studies to confirm the definite superiority of uncut Roux-en-Y anastomosis for patients after distal gastrectomy. Therefore, we propose a multicenter, prospective, randomized clinical trial primarily to elucidate the safety of uncut Roux-en-Y anastomosis compared to Billroth II anastomosis and to innovatively evaluate patients’ nutritional status and quality of life. The SPIRIT 2013 checklist provided the recommended items to address in a clinical trial protocol (shown in Additional file 2).

Moreover, we plan to do some subgroup analysis using the different preoperative nutrition status or the postoperative pathological stages as analysis factors. In our trial, region supervisors will monitor the researchers with regard to completeness of data collection. However, it is unavoidable to have some missing data. As we mentioned in the “Elimination criteria” section, patients whose efficacy evaluation data for the treatment are incomplete will be excluded. Other missing data are divided into two kinds—important data and common data—depending on the relationship of the missing data and the primary or secondary assessment of the trial. We will record and report the number of cases where the randomized treatment cannot be performed. We will try to fix the common data using statistical tools. However, if the missing data are important, we will employ a specialist of statistics to analyze and decide how to deal with them.

However, bias exists in all clinical trials. In our trial, the blinding of the surgeons and patients as to the intervention is the most challenging aspect of designing a randomized controlled trial involving surgical procedures. For others, the blinding of patients should also be considered to reduce potential bias. Therefore, we set purely objective variables as the primary endpoints, including endoscopy and laboratory tests. Additionally, multicenter trials are associated with biases, including differences in operative skill. We have tried to eliminate these biases by examining the surgical procedure by another hospital and training all surgeons at participating centers in standard procedures.

To conclude, based on previous efforts in the reconstruction of distal gastrectomy, the proposed CSGC002 trial represents a multi-center, prospective, randomized controlled trial to test the hypothesis that uncut Roux-en-Y anastomosis can reduce postoperative complications and improve the nutritional status of gastric cancer patients after D2 gastrectomy compared to Billroth II anastomosis. We believe that the results of the trial will significantly contribute to the evidence on which to base the reconstruction of distal gastrectomy.

## Trial status

The total enrollment period is presumed to be 3 years, and the patients will be followed up for 5 years. Patient recruitment commenced on 1 January 2016 and the trial is ongoing.

## Additional files

Additional file 1: : **Table S1.** Gastrointestinal Symptom Rating Scale (GSRS). **Table S2.** Visick classification of upper gastrointestinal symptoms. (DOCX 22 kb)

Additional file 2: SPIRIT 2013 checklist: Recommended items to address in a clinical trial protocol and related documents. (DOC 120 kb)

## Data Availability

Data sharing is not applicable to this article as no datasets were generated or analyzed during the current study.

## Abbreviations

BMI: Body mass index
CRF: Case report form
ECOG: Eastern Cooperative Oncology Group
GSRS: Gastrointestinal Symptom Rating Scale
PNI: Prognosis nutritional index
