# Supplementary material for: Postoperative complications and nutritional status between uncut Roux-en-Y anastomosis and Billroth II anastomosis after D2 distal gastrectomy: a study protocol for a multicenter randomized controlled trial
Source: Trials. 2019 Jul 12;20:428. doi: 10.1186/s13063-019-3531-0 (PMC6626339; doi:10.1186/s13063-019-3531-0)
Supplement: Supplementary file 1 — : Table S1. Gastrointestinal Symptom Rating Scale (GSRS). Table S2. Visick classification of upper gastrointestinal symptoms. (DOCX 22 kb) [file 13063_2019_3531_MOESM1_ESM.docx]

**Table S1 Gastrointestinal Symptom Rating Scale (GSRS)**

| **Parameter** | **Finding** | **Points** |
| --- | --- | --- |
| abdominal pain | no or transient pain | 0 |
|  | occasional aches and pains interfering with some social activities | 1 |
|  | prolonged and troublesome aches and pains causing requests for relief and interfering with many social activities | 2 |
|  | severe or crippling pains with impact on all social activities | 3 |
| heartburn | no or transient heartburn | 0 |
|  | occasional discomfort of short duration | 1 |
|  | frequent episodes of prolonged discomfort; requests for relief | 2 |
|  | continuous discomfort with only transient relief by antacids | 3 |
| acid regurgitation | no or transient regurgitation | 0 |
|  | occasional troublesome regurgitation | 1 |
|  | regurgitation once or twice a day; requests relief | 2 |
|  | regurgitation several times a day; only transient and insignificant relief from antacids | 3 |
| sucking sensation in the epigastrium | no or transient sucking sensation | 0 |
|  | occasional discomfort of short duration; no requests for food or antacids between meals | 1 |
|  | frequent episodes of prolonged discomfort; requests for food and antacids between meals | 2 |
|  | continuous discomfort; frequent requests for food or antacids between meals | 3 |
| nausea and vomiting | no nausea | 0 |
|  | occasional episodes of brief duration | 1 |
|  | frequent and prolonged nausea; no vomiting | 2 |
|  | continuous nausea; frequent vomiting | 3 |
| borborygmus | no or transient borborygmus | 0 |
|  | occasional troublesome borborygmus of short duration | 1 |
|  | frequent and prolonged episodes which can be mastered by moving without impairing social performance | 2 |
|  | continuous borborygmus severely interfering with social performance | 3 |
| abdominal distension | no or transient distension | 0 |
|  | occasional discomfort of short duration | 1 |
|  | frequent and prolonged episodes which can be mastered by adjusting the clothing | 2 |
|  | continuous discomfort seriously interfering with social performance | 3 |
| eructation | no or transient eructation | 0 |
|  | occasional troublesome eructation | 1 |
|  | frequent episodes interfering with some social activities | 2 |
|  | frequent episodes seriously interfering with social performance | 3 |
| increased flatus | no increased flatus | 0 |
|  | occasional discomfort of short duration | 1 |
|  | frequent and prolonged episodes interfering with some social activities | 2 |
|  | frequent episodes seriously interfering with social performance | 3 |
| decreased passage of stools | no or transient | 0 |
|  | every third day | 1 |
|  | every fifth day | 2 |
|  | every seventh day or less frequently | 3 |
| increased passage of stools | once a day | 0 |
|  | 3 times a day | 1 |
|  | 5 times a day | 2 |
|  | 7 times a day or more frequently | 3 |
| loose stools | normal consistency | 0 |
|  | somewhat loose | 1 |
|  | runny | 2 |
|  | watery | 3 |
| hard stools | normal consistency | 0 |
|  | somewhat hard | 1 |
|  | hard | 2 |
|  | hard and fragmented, sometimes in combination with diarrhea | 3 |
| urgent need for defecation | normal control | 0 |
|  | occasional feelings of urgent need for defecation | 1 |
|  | frequent feelings of urgent need for defecation with sudden need for a toilet interfering with social performance | 2 |
|  | inability to control defecation | 3 |
| feeling of incomplete evacuation | feeling of complete evacuation without straining | 0 |
|  | defecation somewhat difficult; occasional feeling of incomplete evacuation | 1 |
|  | defecation definitely difficult; often feelings of incomplete evacuation | 2 |
|  | defecation extremely difficult; regular feelings of incomplete evacuation | 3 |

**Table S2 Visick classification of upper gastrointestinal symptoms**

| Grade | Characteristics |
| --- | --- |
| I | Asymptomatic |
| II | Mild symptoms. Quality of life unaffected. No medication needed. |
| III | Moderate symptoms. Quality of life unaffected. Medication required. |
| IV | Recurrent, incapacitating symptoms equal or worse to pre-operative situation. |
